# Neurodevelopmental trajectories, polygenic risk, and lipometabolism in vulnerability and resilience to schizophrenia

**DOI:** 10.1186/s12888-023-04597-z

**Published:** 2023-03-09

**Authors:** Jia Duan, Xiaohong Gong, Fay Y. Womer, Kaijin Sun, Lili Tang, Juan Liu, Junjie Zheng, Yue Zhu, Yanqing Tang, Xizhe Zhang, Fei Wang

**Affiliations:** 1 Department of Psychiatry. Early Intervention Unit, Affiliated Nanjing Brain Hospital, Nanjing Medical University, Nanjing, 210000 Jiangsu PR China; 2 Department of Psychiatry and Gerontology, The First Affiliated Hospital, China Medical University, 155 Nanjing North Street, Shenyang, 110001 Liaoning PR China; 3 State Key Laboratory of Genetic Engineering and Human Phenome Institute, School of Life Sciences, Fudan University, Shanghai, China; 4 Dept of Psychiatry and Behavioral Sciences, Vanderbilt University Medical Center, Nashville, TN USA; 5 School of Biomedical Engineering and Informatics, Nanjing Medical University, Nanjing, 210000 Jiangsu PR China

**Keywords:** Schizophrenia, Genetic high risk, MRI, Neurodevelopmental trajectories, Polygenic Risk, Lipometabolism

## Abstract

**Background:**

Schizophrenia (SZ) arises from a complex interplay involving genetic and molecular factors. Early intervention of SZ hinges upon understanding its vulnerability and resiliency factors in study of SZ and genetic high risk for SZ (GHR).

**Methods:**

Herein, using integrative and multimodal strategies, we first performed a longitudinal study of neural function as measured by amplitude of low frequency function (ALFF) in 21 SZ, 26 GHR, and 39 healthy controls to characterize neurodevelopmental trajectories of SZ and GHR. Then, we examined the relationship between polygenic risk score for SZ (SZ-PRS), lipid metabolism, and ALFF in 78 SZ, and 75 GHR in cross-sectional design to understand its genetic and molecular substrates.

**Results:**

Across time, SZ and GHR diverge in ALFF alterations of the left medial orbital frontal cortex (MOF). At baseline, both SZ and GHR had increased left MOF ALFF compared to HC (*P* < 0.05). At follow-up, increased ALFF persisted in SZ, yet normalized in GHR. Further, membrane genes and lipid species for cell membranes predicted left MOF ALFF in SZ; whereas in GHR, fatty acids best predicted and were negatively correlated (*r* = -0.302, *P* < 0.05) with left MOF.

**Conclusions:**

Our findings implicate divergence in ALFF alteration in left MOF between SZ and GHR with disease progression, reflecting vulnerability and resiliency to SZ. They also indicate different influences of membrane genes and lipid metabolism on left MOF ALFF in SZ and GHR, which have important implications for understanding mechanisms underlying vulnerability and resiliency in SZ and contribute to translational efforts for early intervention.

**Supplementary Information:**

The online version contains supplementary material available at 10.1186/s12888-023-04597-z.

## Introduction

Schizophrenia (SZ) manifests from a complex interplay of genes and molecules that shape brain structure and function across the lifespan. It appears to be a highly heritable neurodevelopmental disorder [1, 2] with progression of neural alterations in early development leading to psychotic onset usually following puberty. Understanding the vulnerability and resilency factors in SZ is critical for early intervention of this debilitating illness. Studies of SZ and nonpsychotic individuals at genetic high risk for SZ (GHR) and longitudinal designs are especially important for elucidating vulnerability and resiliency to SZ and preventive interventions [3]. GHR individuals, which are defined as those with first-degree relatives with SZ, have almost a tenfold increased risk of developing SZ [4]. Similarities in neural alterations between SZ and GHR have been observed in early development [5–7]. However, prior studies indicate that most GHR do not develop SZ in their lifetime and early neurodevelopmental similarities normalize with age in GHR but not in SZ [7–10]. These studies implicate resiliency factors in GHR and suggest divergent neurodevelopmental trajectories between SZ and GHR.

Longitudinal neuroimaging studies is of increasing importance in characterizing neural trajectory. However, there are significant challenges in performing longitudinal studies in human subjects including participant attrition over time, feasibility of multimodal data collection at every timepoint, and sufficient funding to sustain such study. Some of these challenges could be mitigated by cross-sectional studies that consist of large sample sizes and multimodal data (e.g., genetic and metabolic) collection allowing integrative analysis. Further, previous studies implicate both genetic and metabolic factors in SZ etiology and pathophysiology [11, 12]. Along with neuroimaging, genomic and metabolomic data may provide a more comprehensive perspective on the development of SZ and related vulnerability and resiliency factors.

Genome-wide association studies (GWAS) offer a powerful approach to understanding the genetic basis for brain alterations. While each individual disease-associated single nucleotide polymorphism (SNP) carries only a subtle increase in SZ risk (with odds ratios in the range of 1.1 to 1.2), the cumulative risk of the various SNPs amount to a measure called the polygenic risk score (PRS), capturing the polygenic nature of complex disorders like SZ [13]. Previous studies have examined the effect of PRS for SZ (SZ-PRSs) on functional brain alterations of SZ [14–17]. Studies have shown that SZ-PRS could predict mnemonic hippocampal activity [16] and is associated with left dorsolateral prefrontal cortex (PFC) inefficiency of SZ [15]. A family study also suggests that SZ-PRS affects early neurodevelopment and indicates an increased risk of developing the disorder [18]. Therefore, imaging genetics using PRS could lead to better understanding of genetic determinants of neurodevelopmental deficits in SZ [19].

Metabolomics is also a promising technology to understand brain alterations at the molecular level. Lipids are especially important regulators of brain function and are increasingly implicated in neuropsychiatric disease [20]. One study suggested that changes in lipometabolism presented early in the development of SZ [21]. Levels of lipids were correlated with the severity of negative symptoms in SZ [22, 23]. Moreover, lipid abnormalities have been shown in both postmortem brain tissue and peripheral blood of SZ [24, 25]. In addition, an animal study found that lipid washout led to a change in brain structure [26]. Altogether, these findings demonstrate the importance of lipometabolism in brain alterations associated with SZ.

SZ is a multifaceted disorder arising from a complex interplay of genetic and molecular factors. Thus, integrative and multimodal strategies are needed to comprehensively understand SZ development and pathophysiology. Amplitude of low frequency fluctuations (ALFF), an efficient index of local spontaneous neuronal activity at rest [27], is used to examine neural function. ALFF exhibits moderate to substantial test–retest reliability [28] ensuring a high upper bound for its validity. Evidence has suggested ALFF alterations both in SZ and GHR [29, 30], reflecting their altered neuronal function. In this study, we first performed a longitudinal neuroimaging study of ALFF in SZ and GHR to characterize neurodevelopmental trajectories of SZ and GHR. Using divergent ALFF alterations in SZ and GHR across time, we then examined the influence of polygenic risk and lipometabolism on neural function in SZ and GHR in cross-sectional designs to comprehensively understand genetic and molecular substrates of altered neural function.

## Materials and methods

### Participants

The study was approved by the Medical Science Research Ethics Committee of the First Affiliated Hospital of China Medical University (approval reference number [2012]25–1). All procedures performed in studies involving human participants were in accordance with the ethical standards of the institutional and/or national research committee and with the 1964 Helsinki declaration and its later amendments or comparable ethical standards. All participants provided written informed consent by themselves or by their parents/guardians if they were under 18 years old after a complete description of the study. SZ and GHR participants were recruited from the inpatient and outpatient services at Shenyang Mental Health Center and the Department of Psychiatry at First Affiliated Hospital of China Medical University. Healthy controls (HC) participants were recruited from the local community by advertisement.

All components of the study were conducted at a single site and included both longitudinal and cross-sectional study cohorts, aged 13–45 years. All participants were evaluated by 2 trained psychiatrists to determine the presence or absence of Axis I psychiatric diagnoses using the Structured Clinical Interview for Diagnostic and Statistical Manual of Mental Disorders-IV-Text Revision (DSM-IV) Axis I Disorders (SCID) in those 18 years old and older and the Schedule for Affective Disorders and Schizophrenia for School-Age Children-present and Lifetime Version (K-SADS-PL) in those younger than 18 years. SZ participants met DSM-IV diagnostic criteria for SZ and not any other Axis I disorder. GHR participants were first-degree relatives of individuals with SZ and did not meet criteria for any DSM-IV Axis I disorder. HC participants did not have current or lifetime Axis I disorder or history of psychotic, mood, or other Axis I disorders in first-degree relatives as determined by detailed family history. Participants were excluded if any of the following were present: (1) the existence of substance/alcohol abuse or dependence or concomitant major medical disorder, (2) any magnetic resonance imaging (MRI) contraindications, and (3) history of head trauma with loss of consciousness for ≥ 5 min or any neurological disorder. Symptom severity was measured using the Brief Psychiatric Rating Scale (BPRS).

#### Longitudinal ALFF alterations

Participants were recruited and participated between April 2011 and April 2019. A total of 86 participants were included in this portion of the study, including 21 SZ, 26 GHR, and 39 HC. At baseline, the mean age was 25.93 (7.65) years, aged 13–45 years. There were no significant differences in age among the SZ, GHR, and HC groups; however significant differences were observed in sex (χ^2^ = 12.82, *p* = 0.002). All participants underwent clinical and resting state functional MRI (R-fMRI) assessment at baseline and follow-up at least 10 months after initial scan (ranged 11–67 months). The mean duration between baseline and follow-up scans was 26.16 (14.28) months. Diagnoses were confirmed again at follow-up (using SCID or K-SADS-PL) with no diagnostic change for any participants.

#### The influence of polygenic risk and lipometabolism on ALFF alterations

A total of 298 participants, consisting of 78 SZ, 75 GHR, and 145 HC, were included in this portion of the study. Baseline measures for 51 participants from the longitudinal study were included for this portion. Blood samples were obtained within 24 h of scanning. All participants underwent venipuncture between 10:00 a.m. and 3 p.m. Venous blood samples were centrifuged at 2,000 rpm for 10 min, and then stored at -80 °C for genotyping and lipidomic measurements.

### MRI Acquisition and Data Processing

#### MRI data acquisition

MRI data were acquired using a GE signa HDX 3.0 T scanner (General Electric, Milwaukee, USA) with a standard 8-channel head coil at the First Affiliated Hospital of China Medical University, Shenyang, China. Functional images were collected with a gradient-echo planar imaging (EPI-GRE) sequence. The parameters were as follows: TR = 2000 ms, TE = 30 ms, flip angle = 90°, field of view = 240 × 240 mm^2^, matrix = 64 × 64. Thirty-five axial slices were collected with 3 mm thickness without gap. The scan lasted for 6 min and 40 s, resulting in 200 volumes. Participants were instructed to rest and relax with their eyes closed but remain awake during scanning.

#### Data preprocessing and ALFF calculation

Preprocessing of all functional images was performed using SPM12 (www.fil.ion.ucl.ac.uk/spm/) and DPARSF [31]. The first 10 time points were discarded for magnetic field stabilization and allowing participants to adapt to the scanning environment. The subsequent preprocessing steps included slice time correction and head motion correction. Each participant’s motion was assessed by means of translation/rotation, and an exclusion criterion (translation > 3 mm, rotation > 3° in each direction) was set. Next, the corrected functional images were normalized to MNI space using the EPI template in SPM12, resampled to 3 mm isotropic voxels, and further smoothed via a Gaussian kernel with a 6 mm full-width at half-maximum. Linear detrending was also performed. Finally, voxel-wise ALFF (0.01–0.08 Hz) maps were calculated for each participant. ALFF (0.01–0.08 Hz) of the BOLD signal, which is considered to be physiologically meaningful and related to regional spontaneous neural activity [32], was used to identify regional cerebral function.

### Polygenic risk scores (PRS)

#### Genotyping and quality control

Venous blood was collected from all the participants of cross-sectional study, and genomic DNA was extracted from whole blood according to standard procedures. DNA samples were genome-wide genotyped using the Illumina Global Screening Array-24 v1.0 BeadChip, which provides data for 642,824 fixed genetic variants, in addition to 53,411 customized variants. The quality control parameters used for the exclusion of SNPs were as follows: (1) SNPs with minor allele frequency (MAF) < 1%, (2) SNPs with call rate < 95%, or (3) deviation of a SNP from Hardy–Weinberg equilibrium *p* < 10^–5^. We then applied the following quality control criteria to exclude participants: (1) individuals with excessive missingness > 5%, (2) gender mismatch, and (3) an estimation of identity-by-descent > 0.90.

#### Imputation and calculation of PRS

This process has been previously described [33]. Briefly, genotype imputation was performed by a commercial imputation engine named GenoImpute. We constructed PRS in our sample using the PRSice software (www.PRSice.info) based on common SNP risk effects derived from summary statistics from the international SZ GWAS results conducted by the Psychiatric Genomics Consortium (PGC-SZ) including 33,426 SZ cases and 32,541 controls. *P*-value-informed clumping was performed with a cutoff of *r*^*2*^ = 0.1 in a 250-kb window in order to account for linkage disequilibrium (LD) among SNPs. PRS were calculated using the following twelve PRSs at different *p* value thresholds (pT) (0.0001, 0.001, 0.01, 0.02, 0.03, 0.04, 0.05, 0.1, 0.2, 0.3, 0.4, and 0.5) for each study participant.

#### Bioinformatics enrichment analyses

All the SNPs in SZ-PRS under a certain *p* value threshold were extracted and mapped to the corresponding genes where they were located based on dbSNP database. A gene list was obtained and uploaded to the online tool DAVID Bioinformatics Resources v6.8 (https://david.ncifcrf.gov/,) [34, 35] for the Gene Ontology (GO) and the Kyoto Encyclopedia of Genes and Genomes (KEGG) [36] pathway analyses. The functions of genes were annotated with three GO terms: biological process (BP), cellular component (CC) and molecular function (MF). Multiple testing corrections were performed with Benjamini method (significance level at 0.05).

### Lipidomics

#### Lipidomics profile acquirement.

Untargeted metabolomics analysis were conducted in this study. The measurement details can be found in Additional file 1, including sample preparation, metabolic profiling data acquisition, and data processing. Lipidomics profiles (lipids and lipid-related metabolites) were selected from untargeted metabolomics data to conduct subsequent statistical analysis.

### Statistical Analyses

#### Longitudinal ALFF alterations

Two-way analysis of variance with a repeated measures design was implemented in SPM with group (SZ, GHR or HC) as a between-subject factor, time (baseline and follow-up) as within-subject factor, and age, gender and inter-MRI interval as covariates. Clusters that had main effects of group, time, and especially group-by-time interaction on ALFF values were identified. Significance was set at voxel-level inference of* P* < 0.05 with Gaussian random field (GRF) correction for cluster-level inference of *P* < 0.05.

For regions with significant group-by-time interaction, ALFF values were extracted for additional *post-hoc* analyses. At baseline and follow-up, general linear model was performed among SZ, GHR and HC, with age and gender as covariates. Baseline to follow‐up comparisons were performed at SZ, GHR or HC by the paired t-test. Statistical significance was set to *P* < 0.05.

#### The influence of polygenic risk and lipometabolism on ALFF alterations

ANOVAs (analyses of variance) or chi-square tests were used to examine participants’ demographic characteristics, clinical characteristics, SZ-PRS and lipometabolism accordingly. Results were considered significant at *P* < 0.05.

For participants in this portion of the study, ALFF values were extracted from the regions with significant group by time interaction in the longitudinal study for regression and correlation analyses with SZ-PRS and lipid profiles.

##### SZ-PRS and ALFF alterations

In SZ and GHR, random forest was used to identify the SZ-PRS that could predict significant ALFF alterations identified in the longitudinal study. Random forest is an ensemble method composed of a number of decision trees for regression prediction. Five-fold cross-validation was performed to validate regression results. Subsequently, the prediction model’s performance was evaluated based on mean absolute error (MAE, the average absolute error between the predicted values and the real value) and Pearson correlation coefficient (the correlation between the predicted ALFF values and the actual values). For each SZ-PRS, we separately trained a prediction model in SZ and GHR to predict ALFF values in regions with significant group-by-time interaction in the longitudinal study. A total of 24 models were trained and evaluated.

To further explore the relationships between predictive SZ-PRSs and significant ALFF alterations, partial correlation analyses, controlling for age and sex, were performed in SZ or GHR. Results were considered statistically significant at *P* < 0.05.

##### Lipometabolism and ALFF alterations

Similar to above, regression analyses were performed to identify the lipids and their metabolites that strongly predicted significant ALFF alterations in SZ and GHR. The lipid profiling consists of 474 lipids or lipid-related metabolites that can be divided into 19 lipid species. For each lipid species, we separately trained a prediction model in SZ and GHR to predict ALFF values in regions with significant group-by-time interaction. A total of 38 models were trained and evaluated. Further details can be found in Additional file 1.

To further explore the relationships between predictive lipid species and significant ALFF alterations, partial correlation analyses were performed in SZ or GHR, with age, gender and BMI as covariates. Results were considered statistically significant at *P* < 0.05.

## Results

### Experimental design

**Fig. 1:**
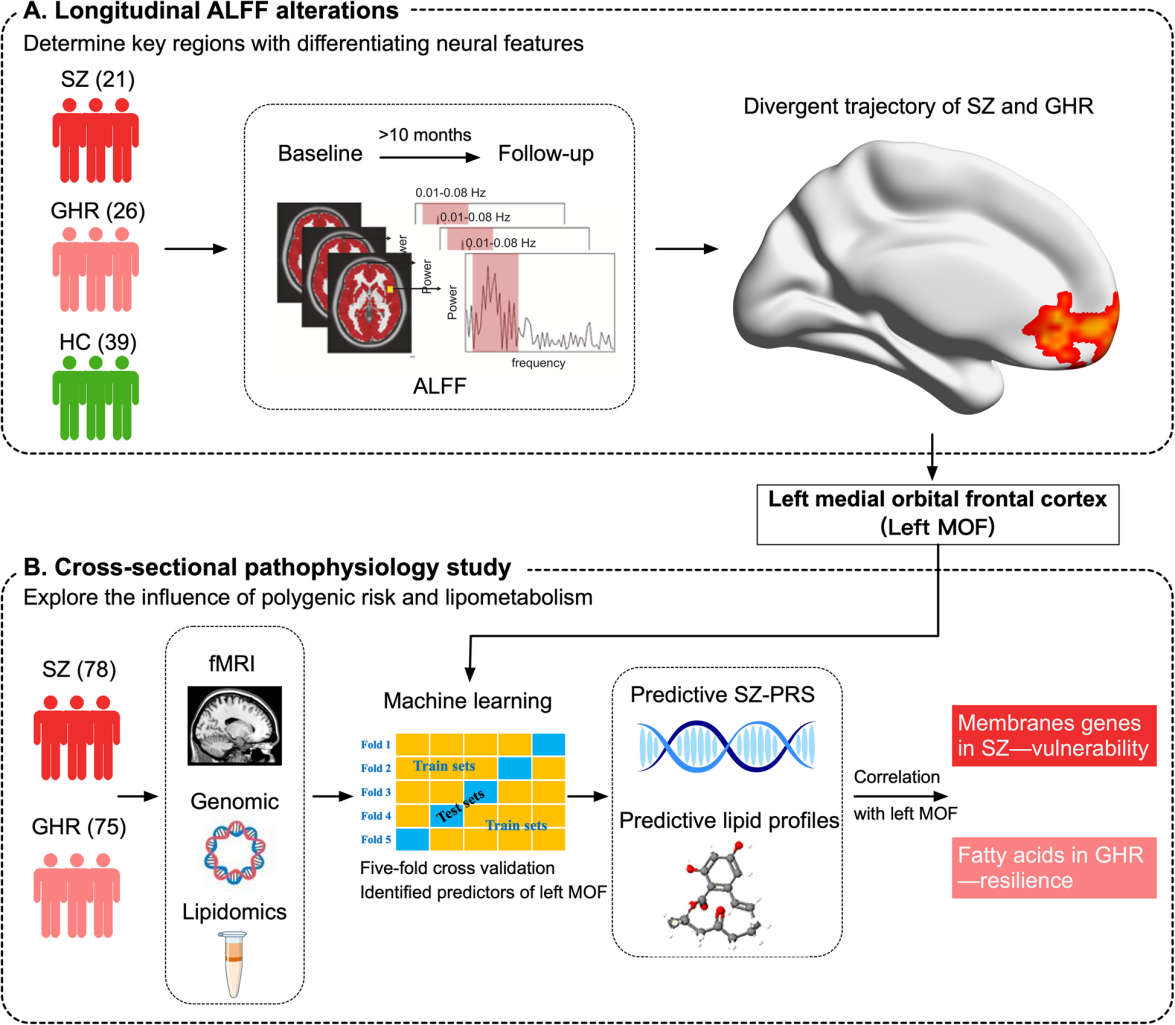
The flowchart of study design. HC, Healthy control; SZ, Schizophrenia; GHR, Genetic high risk; ALFF, Amplitude of low frequency fluctuations; PRS, Polygenic risk scores

We included two parts in this study. First, we performed a longitudinal neuroimaging study of ALFF in SZ and GHR to identify the key regions with differentiating neural features (Fig. 1A). After that, using divergent ALFF alterations in SZ and GHR across time, we then examined the influence of polygenic risk and lipometabolism on neural function and their relationship with neural function in SZ and GHR in cross-sectional designs (Fig. 1B).

### Demographic and Clinical Data

Demographic and clinical information for individuals in the longitudinal study sample is presented in Table 1. There were no significant differences in age among the SZ, GHR, and HC groups at baseline. Significant differences were observed in age at follow-up (F = 3.9, *P* < 0.05, Table 1). Significant differences were observed in gender (χ2 = 12.82, *P* < 0.05, Table 1). Significant differences were observed in BPRS scores among the three groups at baseline and follow-up (Table 1).

**Table 1:** Demographics and clinical characteristics of healthy control, genetic high risk, and schizophrenia in longitudinal study

	**Healthy Control (*n* = 39)**		**Genetic High Risk (*n* = 26)**		**Schizophrenia (*n* = 21)**		**Statistic values**	
	Baseline	Follow-up	Baseline	Follow-up	Baseline	Follow-up	Baseline	Follow-up
**Demographic characteristic**								
Age at scans (year)	27.46 (7.39)	30.59 (7.44)	26 (7.29)	28.38 (6.84)	23 (8.06)	25.05 (7.77)	F = 2.4, *P* = 0.97	F = 3.9, *P* < 0.05
Gender (male/female)	14/25		20/6		7/14		χ^2^ = 12.82, *P* < 0.05	
Duration of MRI interval (month)	29 (14.87)		22.5 (11.48)		25.43 (17.77)		F = 1.68, *P* = 0.19	
**Clinical Characteristics**								
Duration of illness (months)	N/A		N/A		27.0 (38.95)	51.94 (60.27)		
Medication (yes/no)	N/A		N/A		18/3	19/2		
BPRS	18.67 (1.36)	18.77(2.15)	19.46 (2.99)	18.85(1.59)	28.14 (8.07)	23.38(6.60)	F = 31.53, *P* < 0.001	F = 12.47, *P* < 0.001

Data are presented as mean (standard deviation)

*BPRS* Brief Psychiatric Rating Scale

Demographic and clinical information for individuals in the cross-sectional study sample is presented in Table 2. There were significant differences in age among the SZ, GHR, and HC groups (F = 11.18, *P* < 0.001). Significant differences were observed in gender (χ2 = 13.57, *P* = 0.001, Table 2). Significant differences were observed in BPRS scores among the three groups (Table 2).

**Table 2:** Demographics and clinical characteristics of healthy control, genetic high risk, and schizophrenia in cross-sectional study

	**Healthy Control**	**Genetic High Risk**	**Schizophrenia**	F/χ^2^ Values	*P* Values
	(*n* = 145)	(*n* = 75)	(*n* = 78)		
**Demographic characteristic**					
Age at scans (year)	28.28 (7.83)	25.29 (8.24)	23.09 (8.23)	11.18	< 0.001
Gender (male/female)	58/87	43/32	22/56	13.57	0.001
BMI	22.19 (3.63)	22.69 (4.11)	23.39 (4.33)	2.28	0.104
**Clinical characteristic**					
Duration of illness (months)	N/A	N/A	25.05 (40.70)		
First episode (yes/no)	N/A	N/A	55/23		
Medication (yes/no)	N/A	N/A	66/12		
BPRS	*n* = 131	*n* = 72	*n* = 77		
	18.44 (1.03)	18.93 (2.06)	31.73 (10.92)	140.07	< 0.001

Data are presented as mean (standard deviation)

*BMI* Body mass index, *BPRS* Brief Psychiatric Rating Scale

### Longitudinal ALFF alterations

**Fig. 2:**
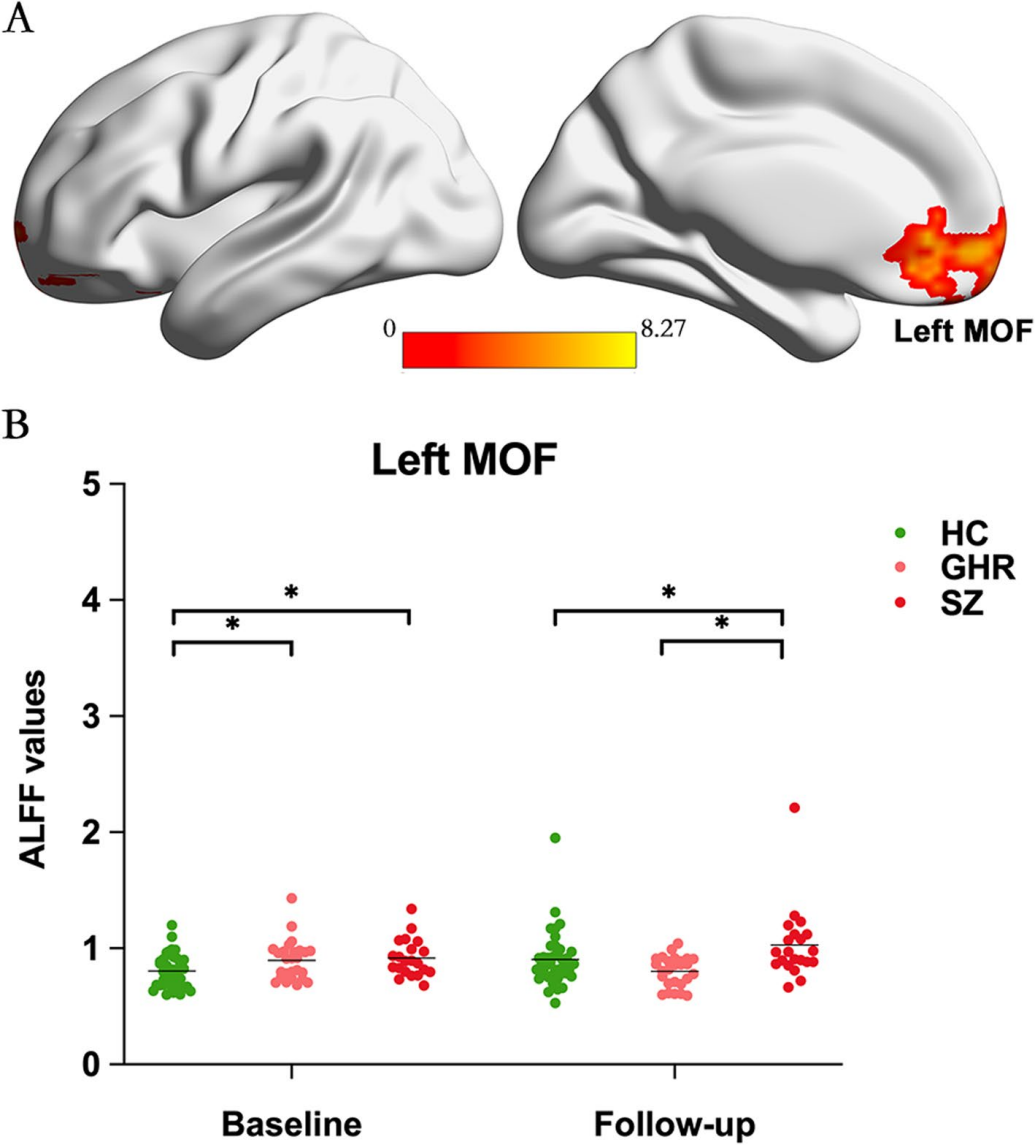
Group-by-time interaction on ALFF among SZ, GHR and HC at baseline and follow-up. **A** Significant group-by-time interaction effect of ALFF values in left MOF (peak at x, y, z = -18, 48, -12; F = 8.27; cluster size = 172). Significance level was set as *p* < 0.05 at voxel level with GRF for multiple comparisons. **B** ALFF values in left MOF of SZ, GHR and HC at baseline and follow-up. The solid lines indicate the mean value. Significance level was set as *p* < 0.05. In SZ, ALFF in left MOF did not significantly change from baseline to follow-up (*P* > 0.05). In GHR, ALFF in left MOF significantly decreased from baseline to follow-up, while ALFF in left MOF significantly increased in HC. HC, Healthy control; SZ, Schizophrenia; GHR, Genetic high risk; MOF, medial orbital frontal cortex; GRF, Gaussian random field correction. *, *p* < 0.05

Group-by-time interaction effect on ALFF was identified in left medial orbital frontal cortex (MOF) (Fig. 2A). Post-hoc analyses revealed that at baseline both SZ and GHR groups had increased ALFF in left MOF compared to HC. At follow-up, compared to HC, increased ALFF in left MOF persisted in SZ but not in GHR (Fig. 2B). Notably, compared to HC, GHR did not significantly differ in ALFF in left MOF at follow-up, but normalized across time (Fig. 2B). In SZ, ALFF in left MOF did not significantly change from baseline to follow-up (*P* > 0.05). In GHR, ALFF in left MOF significantly decreased from baseline to follow-up, while ALFF in left MOF significantly increased in HC.

There were significant main effects of group and time on ALFF (Additional file 1, Figs. S1 and S2).

### The influence of polygenic risk and lipometabolism on ALFF alterations

#### SZ-PRS and ALFF in left MOF

In SZ, the prediction model for SZ-PRS at pT 0.0001 (MAE = 0.153, *r* = 0.25, *P* < 0.05), pT 0.2 (MAE = 0.152, *r* = 0.25, *P* < 0.05) and pT 0.3 (MAE = 0.145, *r* = 0.24, *P* < 0.05) performed relatively better. In GHR, the prediction model of SZ-PRS at pT 0.001 (MAE = 0.121, *r* = 0.31, *P* < 0.05) had relatively better performance. Prediction models for other SZ-PRS had poor performance (*P* > 0.05). SZ-PRS that had better prediction performance were identified as predictive SZ-PRS for SZ and GHR, respectively. Prediction performance for different SZ-PRS are summarized in Additional file 1 (Table S1).

Significant positive correlation was found between left MOF ALFF and predictive SZ-PRS at pT 0.0001 (*r* = 0.349, *P* < 0.005) in SZ. There was no significant correlation between left MOF ALFF and predictive SZ-PRS at pT 0.001 in GHR (*P* > 0.05).

**Fig. 3:**
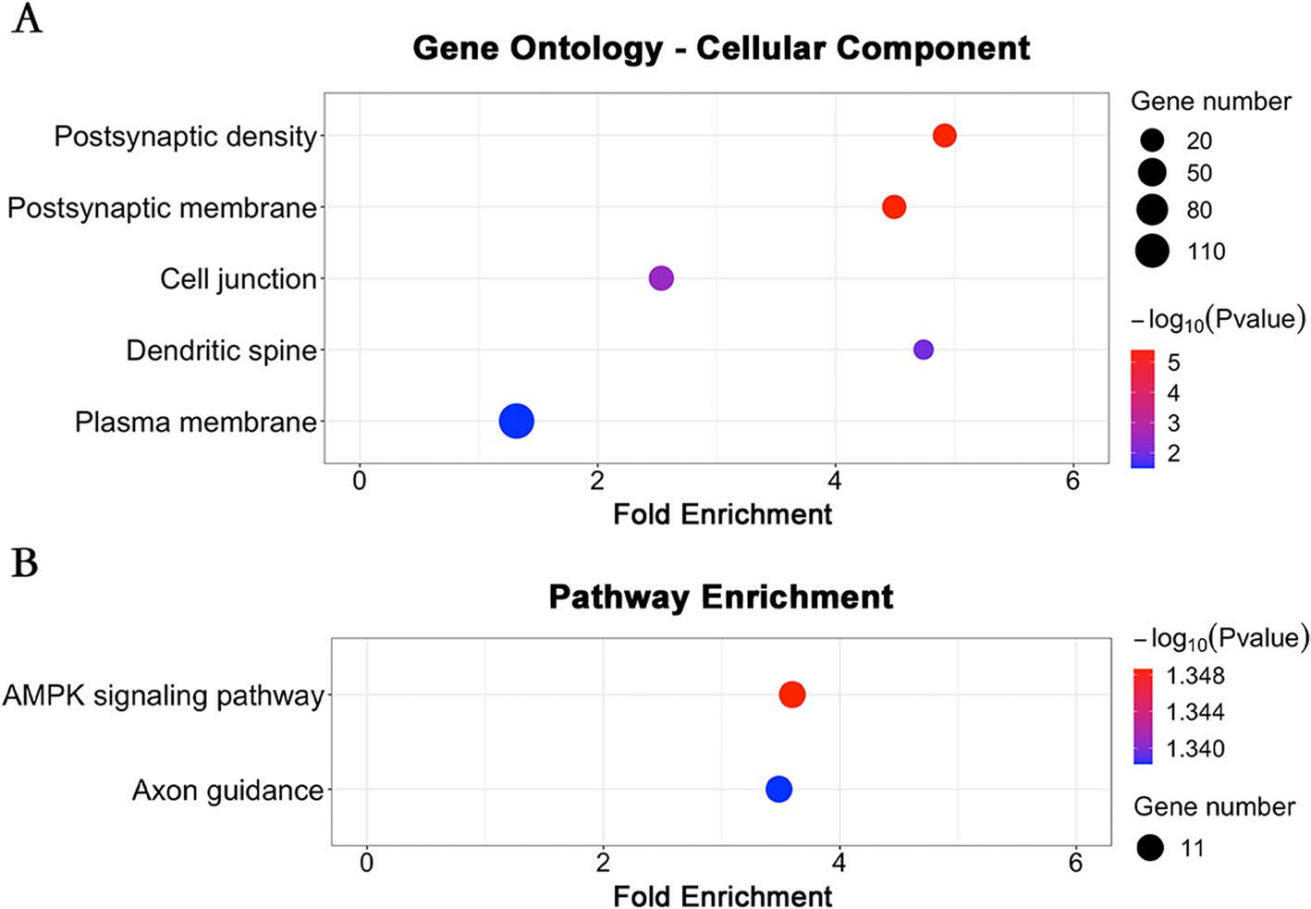
Enrichment analyses for genes of SZ-PRS at pT 0.0001. **A** GO pathway enrichment analyses for genes of SZ-PRS at pT 0.0001. **B** KEGG pathway enrichment analyses for genes of SZ-PRS at pT 0.0001. AMPK, AMP-activated protein kinase

Bioinformatics enrichment analyses were performed for predictive SZ-PRS at pT 0.0001, which was positively correlated with left MOF in SZ as described above. SZ-PRS genes were significantly enriched in five GO terms of cellular component (Fig. 3A), involved in postsynaptic density, postsynaptic membrane, cell junction, dendritic spine and plasma membrane and were mainly located within neuronal structures, particularly cell membranes. Analyses also indicated the significance of two enriched KEGG pathways (Fig. 3B) including AMP-activated protein kinase (AMPK) signaling pathway and axon guidance.

#### Lipometabolism and ALFF in left MOF

In SZ and GHR, all models had good prediction performance (*P* < 0.05). The five lipid species with the least MAE were identified as predictive lipid species for SZ and GHR, respectively. The predictive lipid species for SZ were coenzyme, phosphatidylinositol, lysophosphatidylcholine, sphingomyelin, and ceramide (Table 3). For GHR, these predictive lipid species were fatty acids, ceramide, sphingosines, phosphatidylserine and diglyceride (Table 3). Prediction performance of other lipid species can be found in Additional file 1 (Table S2).

**Table 3:** The five predictive lipid species with the least MAE for left MOF ALFF in SZ or GHR

Lipid species	MAE	*r*	*P*
**Schizophrenia**			
Coenzyme	0.162	0.700	1.77E-08*
Phosphatidylinositol	0.163	0.825	7.06E-14*
Lysophosphatidylcholine	0.165	0.828	9.43E-15*
Sphingomyelin	0.166	0.814	2.03E-13*
Ceramide	0.169	0.827	1.05E-14*
**Genetic High Risk**			
Fatty Acids	0.128	0.853	1.92E-13*
Ceramide	0.129	0.836	4.43E-13*
Sphingosines	0.131	0.817	6.43E-13*
Phosphatidylserine	0.133	0.784	4.56E-11*
Diglyceride	0.138	0.808	7.35E-10*

*MAE* Mean absolute error, *r* Correlation coefficient between the predicted ALFF values and the actual values; *P,* statistical significance of the correlation coefficient

*MOF* Medial orbital frontal cortex

^*^ P < 0.001

**Fig. 4:**
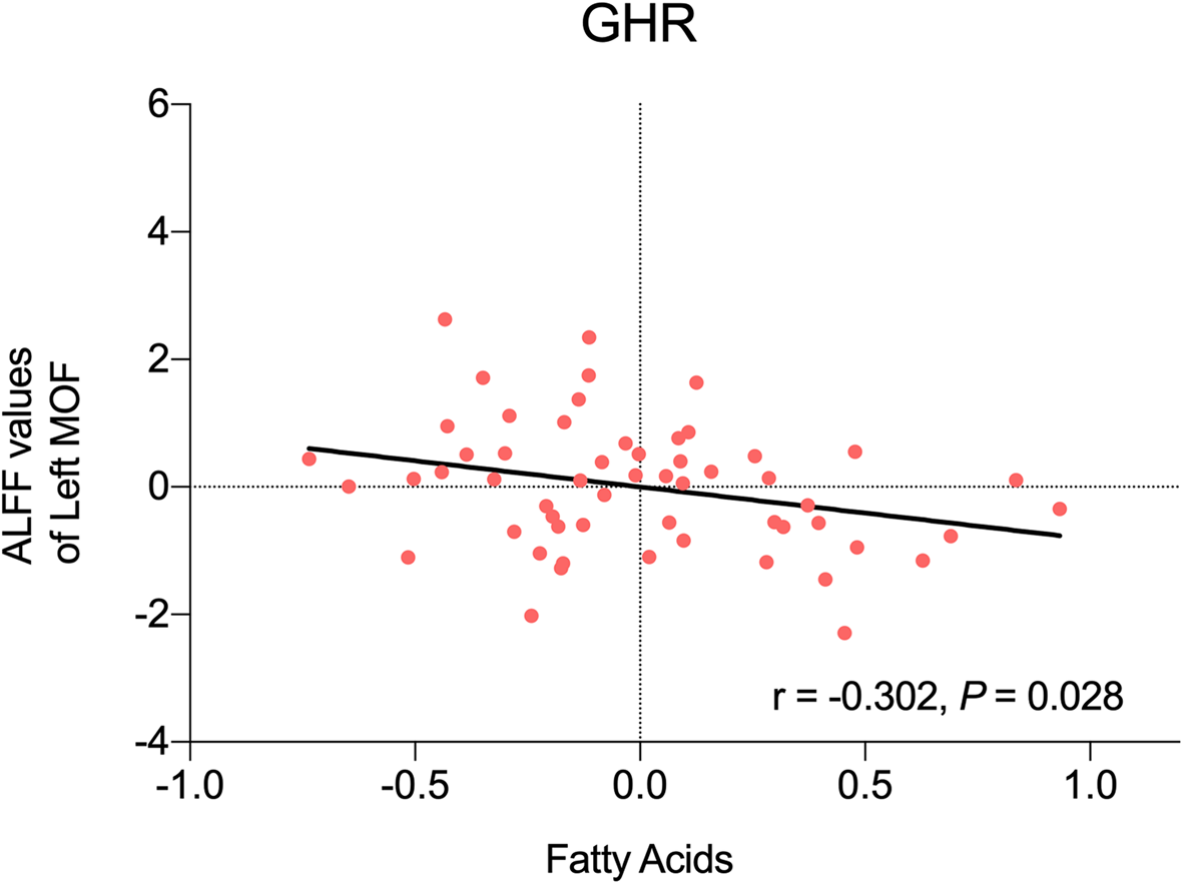
Correlation between ALFF values of left MOF and fatty acids in GHR. GHR, Genetic high risk; MOF, medial orbital frontal cortex

Partial correlation between ALFF values in left MOF and mean lipid levels were performed in SZ and GHR for their respective predictive lipid species. In SZ, there was no significant correlation between ALFF in left MOF and mean levels of these five species (*P* > 0.05). In GHR, left MOF ALFF and mean fatty acid level had significant negative correlation (*r* = -0.302, *P* < 0.05) (Fig. 4).

Significant differences in SZ-PRS and lipometabolism among groups can be found in Additional file 1 (Fig. S4).

## Discussion

In this study, we used a guided and multimodal approach to examine the longitudinal alterations in neural function as measured by ALFF in GHR and SZ and then the influence of polygenic risk and lipometabolism on divergent ALFF alterations in these two groups to understand vulnterability and resiliency to SZ. We first performed a longitudinal study of ALFF alterations in SZ and GHR to determine key regions with differentiating neural features between the two groups. Differentiating features would reflect disease vulnerability that significantly contribute to disease state and resiliency to SZ. Our longitudinal study found that left MOF appears to be a key region in differentiating SZ and nonpsychotic GHR. We then examined the effects of polygenic risk and lipometabolism on left MOF ALFF, as well as their relationship to ALFF in left MOF. In SZ, SZ-PRS at pT 0.0001 predicted left MOF ALFF and had significant positive correlation with it, and bioinformatics enrichment analyses revealed that genes involved in neuron structure and mainly relate to cell membranes may drive this correlation. Correspondingly, lipodomics showed that lipid species involved in cell membranes best predicted left MOF in SZ. In GHR, fatty acids best predicted ALFF in left MOF and had significant negative correlation with left MOF ALFF. These lipid differences may reflect vulnerability and resiliency to SZ. Interestingly, the longitudinal study found normalization of left MOF ALFF in GHR. Taken together with fatty acid findings in GHR, fatty acids may mediate normalization of ALFF in left MOF. Further studies are needed for more definitive conclusions regarding the interplay between polygenic risk, lipometabolism, and left MOF ALFF.

The study of brain development encompasses evaluation of the structural, functional, and network-level changes that occur across the lifespan. Increasing evidence suggested that the brain achieve a maximum level of maturity around the age of 20–30 years, and then begin to decline [37–42]. The longitudinal findings herein support divergent neural trajectories in SZ and GHR during development and across the lifespan. Specifically, the left MOF appears to be a region of divergent ALFF trajectory in SZ and GHR. Left MOF, part of prefrontal cortex (PFC), is thought to contribute to higher cognitive functions [43]. The shared ALFF increases in left MOF in SZ and GHR at baseline could represent shared genetic liability for SZ and may be a neural marker of disease vulnerability. Prefrontal deficits have been previously shown in both SZ and GHR at early ages [10, 44, 45]. Conversely, normalization of ALFF in left MOF at follow-up may reflect resiliency or compensatory mechanisms in GHR individuals who do not develop SZ. Altogether, our findings suggest that left MOF is a critical brain region in understanding the developmental processes leading to SZ.

Consistent with the known heritability of SZ, this study found genetic influences on neural measures in SZ. We found that ALFF in left MOF was positively correlated with predictive SZ-PRS at pT 0.0001 in SZ. This finding is consistent with previous studies in which higher SZ-PRS were associated with functional alterations of PFC [15, 46, 47]. Bioinformatics enrichment analyses indicated that genes involved in neuron structure, especially its cell membranes, likely influence ALFF in left MOF. Many studies report altered neuron structure in the PFC in SZ, including altered postsynaptic density [48], postsynaptic membrane [48], cell junction [49], dendritic spine density [50], and plasma membrane [51] as well as axon guidance [52, 53], in line with our findings. Lipodomic findings further supported the bioinformatics findings. Our analyses showed that phospholipid and ceramide, which are key components of cell membranes, best predicted left MOF ALFF in SZ. Collectively, our findings implicate the importance of cell membrane alterations in the etiology and pathophysiology of SZ.

However in GHR, fatty acids best predicted ALFF in left MOF, different from SZ. Further, fatty acids negatively correlated with left MOF ALFF in GHR. This correlation along with the observed normalization of ALFF in GHR in the longitudinal study suggest fatty acids may contribute to resiliency in GHR. Of note, the lipodomic analyses included baseline measures of a subset of participants in the longitudinal study. Fatty acids play a number of key roles in metabolism, including suppliers of energy and signaling molecules [54]. The passage of fatty acids from the blood to the brain have been previously shown [55]. Reduced levels of fatty acids in plasma and red blood cells have been observed in SZ [23, 56]. Disturbances in PFC fatty acid composition have also been reported in SZ [57, 58], suggesting disturbed PFC fatty acid concentrations as a pathological aspect of SZ. However, little is known about fatty acid alterations in GHR. Nevertheless, there is some evidence to support the role of fatty acids in GHR resilience. Fatty acids have the ability to regulate brain-derived neurotrophic factor (BDNF), which has been associated with the pathophysiology of SZ [59]. Supplementation of fatty acids have been suggested to normalize levels of BDNF and reduce oxidative damage [60], and improve psychiatric symptoms [61]. Altogether, fatty acids may be involved in resiliency in GHR and counteract or compensate for the effects of genetic vulnerability. Further studies are needed.

There are several limitations in this study. Most SZ participants were taking psychotropic medications at the time of the study, and thus there may be confounding effects as GHR participants were not taking psychotropic medications. The longitudinal sample was only of moderate size. Further studies are needed in a larger and unmedicated sample of SZ and GHR to confirm our results. Moreover, gender and age factors were not controlled in the recruitment of participants to ensure the sample size of longitudinal study, which may confound our findings. We therefore performed analysis to test for interactions between gender and group, and age and group. At baseline and follow-up, there was no significant interaction effect of group x gender and group x age on left MOF ALFF in the longitudinal study. There was also no significant interaction effect of group x gender and group x age on the lipids and on left MOF ALFF in the cross-sectional study. In addition, there was no significant linear correlation between ALFF in left MOF and lipid species that best predicted left MOF ALFF in SZ. There also was no significant correlation between left MOF ALFF and predictive SZ-PRSs in GHR. Possible explanations for these findings include complex relationships among polygenic risk, lipometabolism, and ALFF that cannot be simply accounted for by linear correlations. Therefore, further studies are needed to investigate the complex interplay between SZ-PRS, lipids, and ALFF in left MOF in SZ and GHR.

## Conclusions

Altogether, using a guided, multimodal approach, our findings implicate left MOF as a key region with divergent neural trajectory between SZ and GHR. Genomic and lipodomic analyses implicate the importance of membrane genes and lipids in differential ALFF alterations and their progression in left MOF in SZ and GHR, reflecting potential indicators of vulnerability and resiliency to SZ. The findings herein suggest that specific lipids and left MOF function may be important targets for early intervention or prevention in GHR, although further studies are warranted for definitve conclusions. These findings may have important implications for understanding mechanisms underlying vulnerability and resiliency in SZ and contribute to translational efforts for early intervention and prevention in SZ.

## Supplementary Information

**Additional file 1: Figure S1. **Main effects of group on ALFF among SZ, GHR and HC. **Figure S2. **Main effects of time on ALFF among SZ, GHR and HC between baseline and follow-up. **Figure S3. **Group-by-time interaction on ALFF among SZ, GHR and HC in cross-sectional study. **Figure S4.** Significant differences in SZ-PRS among groups. **Table S1. **The prediction results of SZ-PRSs for ALFF values of left MOF in SZ or GHR. **Table S2. **The prediction results of other lipid species for ALFF values of left MOF in SZ or GHR.

## Data Availability

The datasets analyzed in the current study are available from the corresponding author on reasonable request.

## Abbreviations

SZ: Schizophrenia
GHR: Genetic high risk for SZ
ALFF: Amplitude of low frequency function
SZ-PRS: Polygenic risk score for SZ
MOF: Medial orbital frontal cortex
GWAS: Genome-wide association studies
SNP: Single nucleotide polymorphism
PFC: Prefrontal cortex
DSM-IV: Diagnostic and Statistical Manual of Mental Disorders-IV-Text Revision
SCID: Statistical Manual of Mental Disorders
K-SADS-PL: Schedule for Affective Disorders and Schizophrenia for School-Age Children-present and Lifetime Version
MRI: Magnetic resonance imaging
BPRS: Brief Psychiatric Rating Scale
MAF: Minor allele frequency
GO: Gene Ontology
KEGG: Kyoto Encyclopedia of Genes and Genomes
BP: Biological process
CC: Cellular component
MF: Molecular function
GRF: Gaussian random field
ANOVAs: Analyses of variance
MAE: Mean absolute error
