# Supplementary material for: Neurodevelopmental trajectories, polygenic risk, and lipometabolism in vulnerability and resilience to schizophrenia
Source: BMC Psychiatry. 2023 Mar 9;23:153. doi: 10.1186/s12888-023-04597-z (PMC9999573; doi:10.1186/s12888-023-04597-z)
Supplement: Supplementary file 1 — Additional file 1: Figure S1. Main effects of group on ALFF among SZ, GHR and HC. Figure S2. Main effects of time on ALFF among SZ, GHR and HC between baseline and follow-up. Figure S3. Group-by-time interaction on ALFF among SZ, GHR and HC in cross-sectional study. Figure S4. Significant differences in SZ-PRS among groups. Table S1. The prediction results of SZ-PRSs for ALFF values of left MOF in SZ or GHR. Table S2. The prediction results of other lipid species for ALFF values of left MOF in SZ or GHR. [file 12888_2023_4597_MOESM1_ESM.docx]

Additional file

**Untargeted lipidomics and metabolomics**

**Sample Preparation.** Peripheral venous blood samples from all participants were collected in 5ml EDTA Vacutainer tubes containing heparin sodium. Then the samples were centrifuged for 10 min (2000 rpm, 4°C). Each aliquot (150μl) of plasma sample was stored at –80°C until metabolomics analysis.

For lipidomics, 20 μL aliquots of plasma samples were added into 120 μL aliquots of methanol solution containing internal standards (lysophosphatidylcholine17:0 (3.12 μg/mL), triglyceride 45:0 (2 μg/mL) and Ceramide d18:1/26:0/18:1(d9) (1.68 μg/mL, Alabaster, Alabama, USA)). After vortexing for 2 min, 360μL of MTBE and 100μL ultra-pure water were sequentially pipetted into the extracts and vortexed again for homogenous mixing, the resultant extracts were shaken for 15 min under room temperature, standby for 30 min and centrifuged for 15 min under 4 °C. 150 μL aliquots of the extracts were transferred and freeze-dried until subsequent analysis. The dry extracts were reconstituted with 150 aliquots of isopropanol-acetonitrile mixture solution (1:1, v:v) for untargeted lipidomics analyses. Quality control (QC) samples were prepared by mixing equal aliquots（5μL）of each sample, and then inserted regularly and analyzed in every 30 samples for monitoring the stability and repeatability of instrument analysis.

For metabolomics, 110 μL aliquots of plasma samples were added into 440 μL aliquots of metabolite extract solution containing internal standards (phenylalanine-d5 (200 ng/mL), clenbuterol-d9 (200 ng/mL, Andover, Massachusetts, USA), and chloramphenicol (200 ng/mL, Augsburg, Germany)) for precipitating proteins; The mixtures were vortexed for 2 min to extract metabolites. And the supernatants were removed into another collector after centrifuging at 1500 rpm for 15 min. The resultant supernatants from each sample were equally divided into two aliquots and then freezing-dried at -52°C. The extracts were reconstructed with 150 aliquots of methanol-water mixture solution (1:3, v:v) for untargeted metabolomics analyses .

**Metabolic Profiling Data Acquisition.** In untargeted lipidomics analysis, an Accucore C30 column (Thermo Fisher Scientific, USA, 2.6 μm, 2.1 × 100 mm) was employed and eluted with 60% acetonitrile in water (A) and 10% acetonitrile in isopropanol (B) containing 10 mM ammonium formate and 0.1% formic acid. The separation gradient was optimized as follow: initial 10% B maintaining for the beginning 0.5 min, ramping to 50% in 2.5 min, further increasing to 85% in 12 min, and finally reaching 100% B in 13 min, the other 7 min for column washing and equilibration using 0.3 mL/min flowrate. Lipid extracts were profiled with the same parameters as the metabolome used (300-1500 m/z). The key settings were as follows: 70,000 FWHM full scan resolution, 17,500 FWHM. MS/MS resolution, loop count 10, AGC target 3e6, maximum injection time 200 ms and 80 ms for full scan and MS/MS respectively, dynamic exclusion 8s. Stepped normalized collision energy 25%+40% and 35% were employed for positive and negative mode after optimization. All the metabolomics data acquired as profile format by XCalibur workstation (Thermo Scientific, San Jose, USA).

Untargeted metabolomics analysis was performed based on Ultimate 3000 ultra-high performance liquid chromatograph coupled with Q Exactive quadrupole-Orbitrap high resolution mass spectrometer UPLC-HRMS system (Thermo Scientific, San Jose, USA). Chromatographic separation was implemented on Acquity BEH C18 column (Waters Co., Milford, Massachusetts, USA. 1.7 μm, 2.1 × 100 mm) for both positive and negative ionization models. Metabolites were eluted by 0.1% formate/water and acetonitrile using linear gradient ramping from 2% organic mobile phase to 98% in 10 min for positive mode. In negative mode, water and acetonitrile/methanol both containing ammonium bicarbonate buffer salt (pH 9.0 adjusted by ammonium hydroxide) were employed to elute metabolites. The gradient was used as follow: 0 min 2% organic phase ramped to 100% in 10 min, and other 5 min was used for column washing and equilibrating. Data acquisition was performed with 70~1000 m/z in positive mode and 80~1000 m/z in negative mode. Parameters of mass spectrometry were as follows: ionization voltage, 4000V for positive mode, 3500 V for negative mode, aux gas 10 arb, heater temperature 350℃, capillary temperature 320℃ and S-Lens RF level 50%. The metabolite extracts were profiled with full scan mode under 70,000 FWHM resolution with AGC 3e6 and 200 ms maximum injection time. 70,000 and 17,500 FWHM were used for full scan and MS/MS data acquisition, respectively. Apex trigger, dynamic exclusion and isotope exclusion was turned on, precursor isolation window as set at 1.0 Da. 15%, 30% and 45% stepped normalized collision energy was employed for collision induced disassociation of metabolite using ultra-pure nitrogen as fragmentation gas.

**Data Processing.** Untargeted lipidomics data was processed with LipidSearch software including peak picking and lipid identification. The acquired MS2 spectra were searched against in silico predicted spectra. The mass accuracy for precursor and MS/MS product ions searching were 5 ppm and 5 mDa, respectively. The lipid identification was checked and investigated one-by-one to eliminate false positive chiefly basing on peak shake, adduct ions behavior, fragmentation pattern, and chromatographic behavior. And the area under curve values (AUC) for all the lipid molecules were extracted as quantitative information of lipids with TraceFinder software version 4.1 (Thermo Scientific, USA). Finally, all peak area data for annotated metabolites and AUC data for annotated lipid molecules were exported into Excel software (Microsoft, USA) followed by further data normalization with linear regression (Loreg) algorithm2.

For raw metabolomics data, Peak-alignment, peak-identification and peak-extraction were performed using Compound Discoverer version 2.1 and TraceFinder software version 4.1 (Thermo Scientific, USA). Metabolite structural annotation was conducted through searching against Human Metabolome Database (HMDB), Kyoto Encyclopedia of Genes and Genomes (KEGG) and a self-built MS/MS spectrum library created using authentic standards as well as mzCloud spectral library (mzCloud.org). For metabolite identification, mass accuracy of precursor within ± 5 ppm was prerequisite, meanwhile, isotopic information including at least 1 isotope within 10 ppm and fit score of relative isotopic abundance pattern 70% were introduced to confirm the chemical formula in addition to exact mass.

**SZ-PRS and lipids importance evaluation**

Before training the model, some preprocessing steps was adopted. First, mean filling method was used to process the missing values of BMI, which using average value of the column where the missing value is located for data filling. Then, z-score was used to standardize the data so that the processed data conforms to the standard normal distribution. At last, we use nuisance regression to eliminate the influence of covariate, including gender, age and BMI (BMI for lipids, not for SZ-PRS). Random forest was used as regression model. Random forest is composed of a number of decision trees, an ensemble method to making regression prediction. The number of decision trees in our model is 50, the max depth of each decision tree is set to 4, the minimum number of samples that the subtree can continue to be divided is set to 2, and maximum number of features in decision tree division is the sqrt of the number of data features.

**Main effects of group and time**

Significant main effects of group were found in ALFF values of bilateral middle frontal gyrus, bilateral superior medial frontal gyrus, bilateral postcentral gyrus, bilateral calcarine, bilateral lingual gyrus, bilateral precuneus, left angular gyrus, left inferior parietal (Figure S1).

Significant main effects of time were found in left inferior temporal gyrus, left middle temporal gyrus, bilateral rectus gyrus, left inferior frontal gyrus (orbital part), left middle temporal gyrus, left middle occipital gyrus, left precuneus, bilateral median cingulate gyrus (Figure S2).

**ALFF value of left MOF in Cross-Sectional Analyses**

SZ and GHR had increased ALFF in the left MOF in Cross-Sectional analyses compared with HC (Figure S3).

**Significant differences in SZ-PRS and lipometabolism among groups**

There were significant differences of SZ-PRS at pT 0.0001, pT 0.01, pT0.1 and pT 1 among groups. Post-hoc analysis showed that all four SZ-PRS had increased in SZ, compared to HC (*P* < 0.05, Figure S4). There was no significant difference of other SZ-PRS (P > 0.05).

Determined by Kruskal-Wallis non-parametric test for the non-normal distributed metabolomics data, three groups have significantly difference in mean levels of bile acids, acyl carnitine, ceramide, coenzyme, lysophosphatidylcholine, lysophosphatidylethanolamine, phosphatidylethanolamine, phosphatidylinositol, phosphatidylserine, choline. Further post hoc analysis revealed that compared to HC, SZ has decreased mean bile acids and increased mean phosphatidylinositol. Compared to HC, GHR had increased mean ceramide, coenzyme, lysophosphatidylcholine, lysophosphatidylethanolamine, phosphatidylethanolamine, phosphatidylinositol and phosphatidylserine; GHR also had decreased mean choline.

**Predictive Model.**

The prediction results of SZ-PRSs and other lipid species for ALFF values of left MOF were shown in Table S1 and Table S2.

**Figure S1.** Main effects of group on ALFF among SZ, GHR and HC.


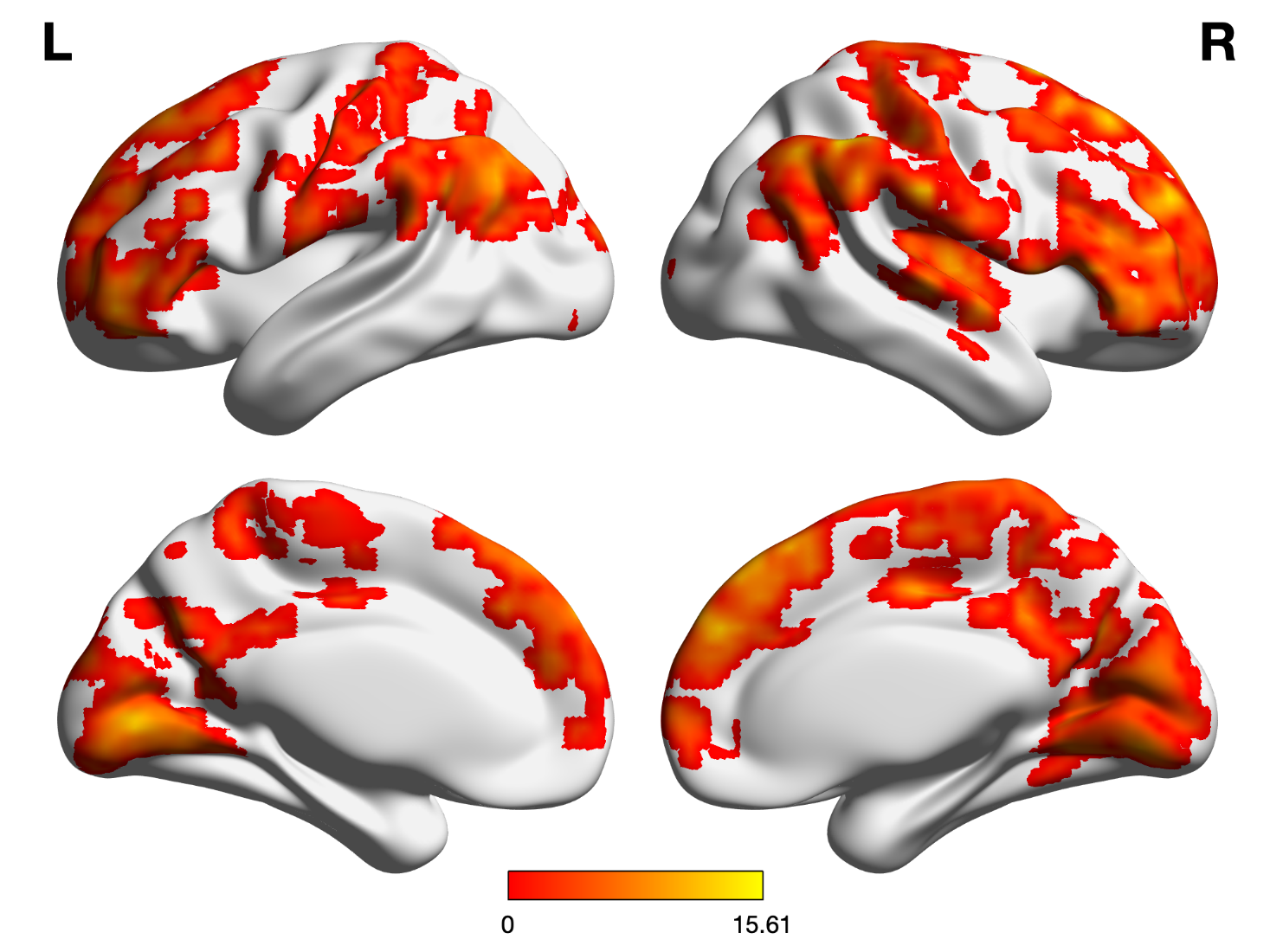


**Fig S1.** Regions showing significant group effects on ALFF among SZ, GHR and HC.

R, Right; L, Left;

**Figure S2.** Main effects of time on ALFF among SZ, GHR and HC between baseline and follow-up.


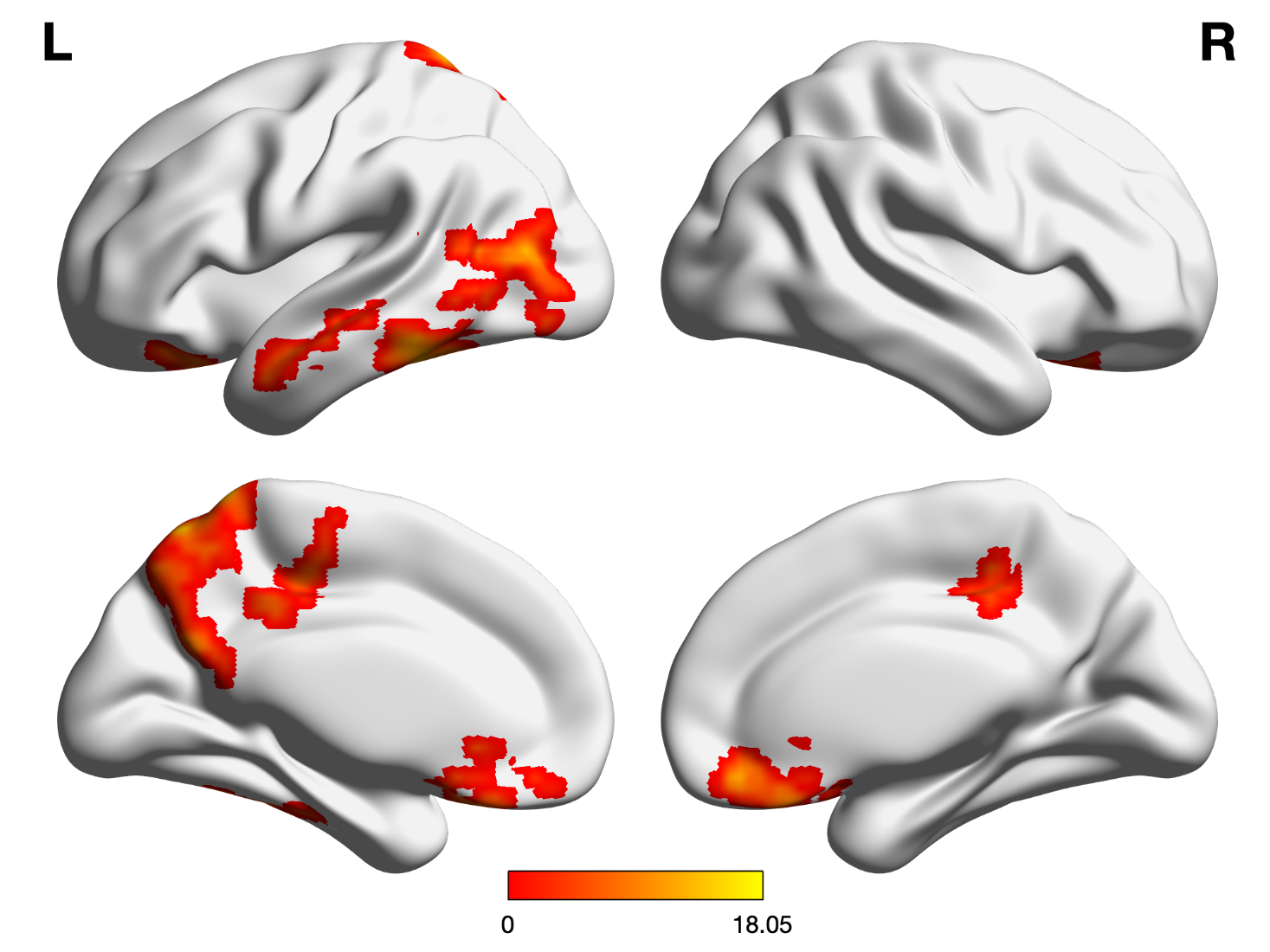


**Fig S2.** Regions showing significant time effects on ALFF among SZ, GHR and HC between baseline and follow-up.

R, Right; L, Left;

**Figure S3.** Group-by-time interaction on ALFF among SZ, GHR and HC in cross-sectional study.


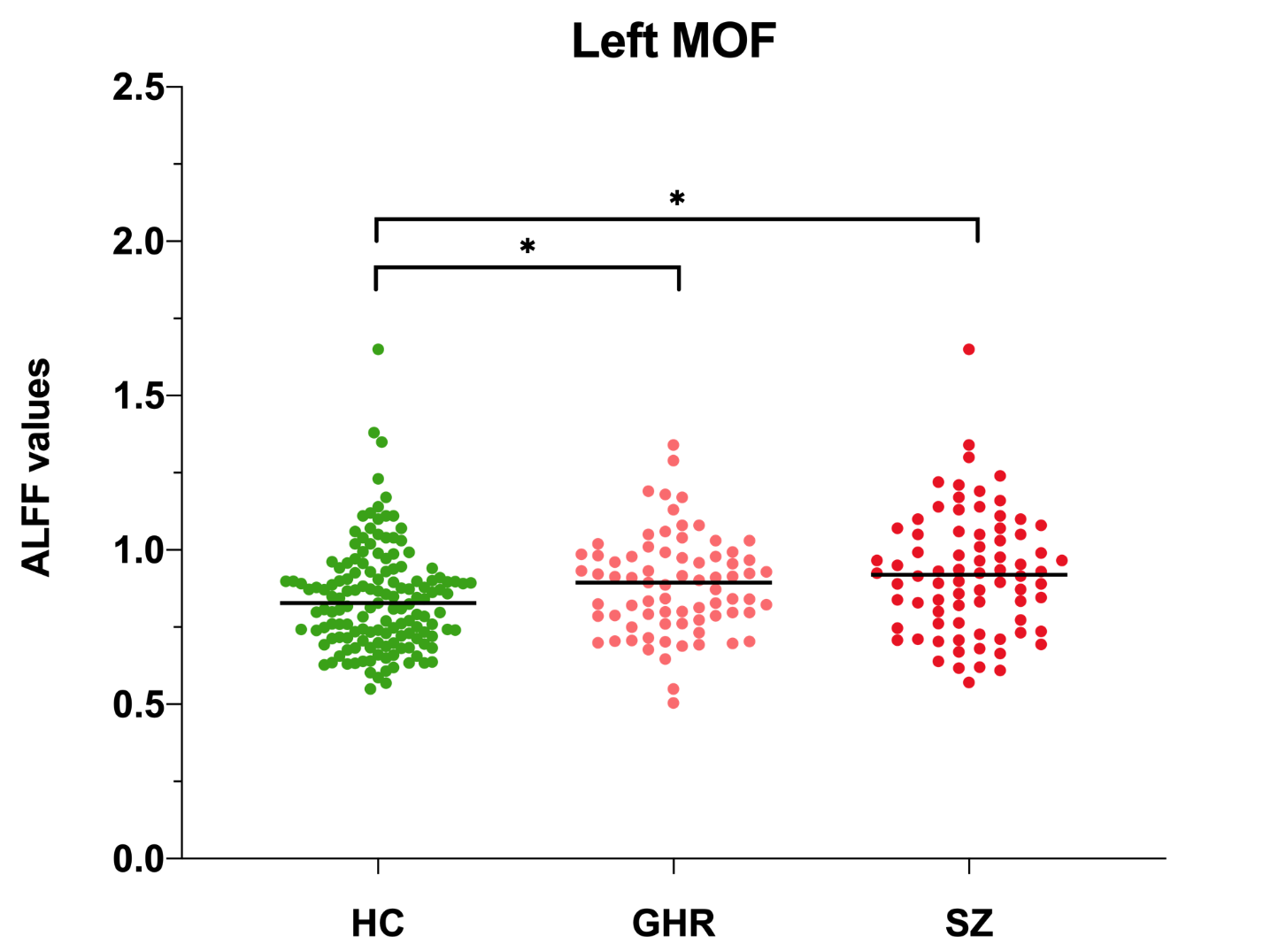


**Fig S3.** ALFF values in left MOF of SZ, GHR and HC in cross-sectional study. The solid lines indicate the mean value. Significance level was set as p < 0.05.

HC, Healthy control; SZ, Schizophrenia; GHR, Genetic high risk; MOF, superior frontal gyrus, medial orbital.

*, p < 0.05

**Figure S4. Significant differences in SZ-PRS among groups.**


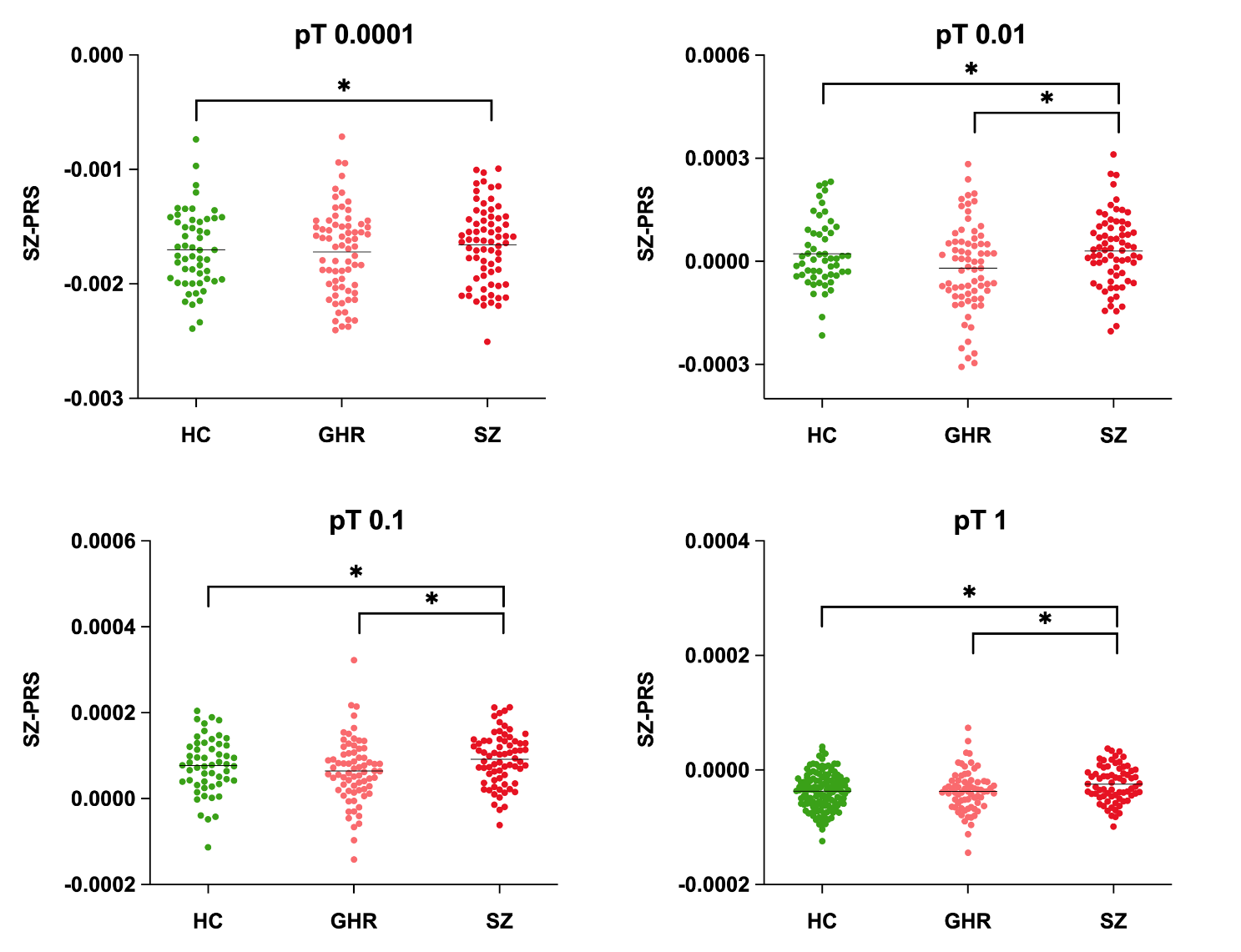


HC, Healthy control; SZ, Schizophrenia; GHR, Genetic high risk.

* *P* < 0.05

**Table S1.** The prediction results of SZ-PRSs for ALFF values of left MOF in SZ or GHR.

| **SZ-PRSs** | **MAE** | ***r*** | ***P*** |
| --- | --- | --- | --- |
| **Schizophrenia** |  |  |  |
| pT_0.0001 | 0.153 | 0.25 | 0.037* |
| pT_0.001 | 0.153 | 0.2 | 0.11 |
| pT_0.01 | 0.165 | -0.25 | 0.036 |
| pT_0.02 | 0.153 | 0.085 | 0.38 |
| pT_0.03 | 0.161 | 0.1 | 0.4 |
| pT_0.04 | 0.155 | 0.089 | 0.46 |
| pT_0.05 | 0.176 | -0.054 | 0.66 |
| pT_0.1 | 0.158 | 0.14 | 0.25 |
| pT_0.2 | 0.152 | 0.25 | 0.034* |
| pT_0.3 | 0.145 | 0.24 | 0.047* |
| pT_0.4 | 0.152 | 0.2 | 0.1 |
| pT_0.5 | 0.157 | 0.19 | 0.11 |
| **Genetic High Risk** |  |  |  |
| pT_0.0001 | 0.124 | 0.13 | 0.29 |
| pT_0.001 | 0.120 | 0.31 | 0.0086* |
| pT_0.01 | 0.130 | -0.077 | 0.52 |
| pT_0.02 | 0.136 | -0.25 | 0.034 |
| pT_0.03 | 0.129 | -0.064 | 0.6 |
| pT_0.04 | 0.150 | -0.052 | 0.67 |
| pT_0.05 | 0.142 | -0.07 | 0.56 |
| pT_0.1 | 0.132 | -0.15 | 0.21 |
| pT_0.2 | 0.137 | 0.005 | 0.97 |
| pT_0.3 | 0.125 | -0.007 | 0.95 |
| pT_0.4 | 0.126 | -0.035 | 0.77 |
| pT_0.5 | 0.127 | -0.027 | 0.83 |

MAE, mean absolute error; *r*, correlation coefficient between the predicted ALFF values and the actual values; *P,* statistical significance of the correlation coefficient.

* *P* < 0.05 and *r* > 0.

**Table S2.** The prediction results of other lipid species for ALFF values of left MOF in SZ or GHR.

| **Lipid species** | **MAE** | ***r*** | ***P*** |
| --- | --- | --- | --- |
| **Schizophrenia** |  |  |  |
| Phosphatidylcholine | 0.170 | 0.814 | 8.37E-12* |
| Sphingosines | 0.172 | 0.750 | 1.29E-09* |
| Phosphatidylethanolamine | 0.173 | 0.794 | 5.34E-10* |
| Triglyceride | 0.174 | 0.833 | 9.65E-13* |
| Acyl Carnitine | 0.176 | 0.773 | 1.71E-12* |
| Lysophosphatidylethanolamine | 0.176 | 0.787 | 2.83E-12* |
| Cholesterol Ester | 0.178 | 0.758 | 5.76E-12* |
| Diglyceride | 0.179 | 0.756 | 1.07E-08* |
| Bile Acids | 0.182 | 0.794 | 3.03E-13* |
| Phosphatidylserine | 0.184 | 0.710 | 9.22E-07* |
| Steroids | 0.184 | 0.770 | 5.31E-09* |
| Carnitines | 0.185 | 0.779 | 1.22E-11* |
| Fatty Acids | 0.193 | 0.774 | 4.84E-12* |
| Choline | 0.197 | 0.740 | 2.94E-10* |
| **Genetic High Risk** |  |  |  |
| Lysophosphatidylethanolamine | 0.140 | 0.771 | 6.46E-10* |
| Cholesterol Ester | 0.141 | 0.765 | 4.19E-09* |
| Coenzyme | 0.141 | 0.660 | 2.47E-07* |
| Acyl Carnitine | 0.142 | 0.792 | 5.96E-10* |
| Lysophosphatidylcholine | 0.143 | 0.795 | 3.86E-11* |
| Carnitines | 0.145 | 0.806 | 5.52E-12* |
| Bile Acids | 0.145 | 0.784 | 2.37E-09* |
| Triglyceride | 0.146 | 0.810 | 8.44E-13* |
| Phosphatidylcholine | 0.146 | 0.786 | 6.68E-11* |
| Choline | 0.147 | 0.756 | 8.00E-09* |
| Phosphatidylinositol | 0.148 | 0.760 | 3.75E-10* |
| Sphingomyelin | 0.148 | 0.762 | 5.31E-10* |
| Steroids | 0.149 | 0.767 | 1.27E-10* |
| Phosphatidylethanolamine | 0.149 | 0.785 | 4.29E-08* |

MAE, mean absolute error; *r*, correlation coefficient between the predicted ALFF values and the actual values; *P,* statistical significance of the correlation coefficient.

* *P* < 0.001
